# *WT1* expression in breast cancer disrupts the epithelial/mesenchymal balance of tumour cells and correlates with the metabolic response to docetaxel

**DOI:** 10.1038/srep45255

**Published:** 2017-03-27

**Authors:** Mara Artibani, Andrew H. Sims, Joan Slight, Stuart Aitken, Anna Thornburn, Morwenna Muir, Valerie G. Brunton, Jorge Del-Pozo, Linda R. Morrison, Elad Katz, Nicholas D. Hastie, Peter Hohenstein

**Affiliations:** 1 Institute of Genetics and Molecular Medicine, University of Edinburgh, Crewe Road South, Edinburgh, EH4 2XU, UK; 2 Royal (Dick) School of Veterinary Studies, University of Edinburgh, Easter Bush Campus, Midlothian, EH25 9RG, UK; 3 The Roslin Institute, University of Edinburgh, Easter Bush Campus, Midlothian, EH25 9RG, UK.

## Abstract

WT1 is a transcription factor which regulates the epithelial-mesenchymal balance during embryonic development and, if mutated, can lead to the formation of Wilms’ tumour, the most common paediatric kidney cancer. Its expression has also been reported in several adult tumour types, including breast cancer, and usually correlates with poor outcome. However, published data is inconsistent and the role of WT1 in this malignancy remains unclear. Here we provide a complete study of *WT1* expression across different breast cancer subtypes as well as isoform specific expression analysis. Using *in vitro* cell lines, clinical samples and publicly available gene expression datasets, we demonstrate that WT1 plays a role in regulating the epithelial-mesenchymal balance of breast cancer cells and that *WT1*-expressing tumours are mainly associated with a mesenchymal phenotype. *WT1* gene expression also correlates with *CYP3A4* levels and is associated with poorer response to taxane treatment. Our work is the first to demonstrate that the known association between WT1 expression in breast cancer and poor prognosis is potentially due to cancer-related epithelial-to-mesenchymal transition (EMT) and poor chemotherapy response.

The Wilms’ Tumour gene 1 (*WT1*), encodes for a complex protein with transcription factor activity which is essential in mammals throughout life^1^. Its roles vary with the developmental stages: in the embryo, it regulates the epithelial-mesenchymal balance required for a correct organogenesis and acts as a tumour suppressor in the aetiology of Wilms’ tumour^2^,^3^; in the adult, it is involved in the maintenance of tissue homeostasis and has been controversially considered as an oncogene in leukaemia and several solid tumours, including breast cancer^4^. The role of *WT1* in this malignancy is unclear, despite the accumulation of a considerable body of data over the last fifteen years. Several groups have reported *WT1* expression in breast cancer, but the data on the percentage of WT1-positive tumours are highly discordant, most likely because of differences in the detection method, antibody specificity and histological subtype^5^,^6^,^7^,^8^,^9^,^10^,^11^. Moreover, there is evidence for both oncogenic and tumour suppressing functions^6^,^7^,^12^ and these contradictory results can be explained only in part by the presence of different isoforms^13^.

One study has associated WT1 with improved outcomes^14^, however most literature seems to indicate that high WT1 expression is associated with poor prognosis in breast cancer patients^8^,^15^. The biological basis behind these seemingly inconsistent findings has not yet been identified, but several hypotheses have been formulated. Firstly, tumours expressing high levels of WT1 may have a proliferative advantage since silencing WT1 leads to growth arrest and apoptosis^16^,^17^,^18^,^19^. Investigations on the molecular mechanisms involved in this process have revealed that WT1 can modulate many genes of the *BCL2* family, including *BAK, BAG3, BCL2A1* and *BCL2* itself 20,21,22 as well as regulate the Fas-related death signalling pathway^23^; moreover, there is some evidence suggesting that WT1 can promote cell proliferation by up-regulating cyclin D1^24^.

A second hypothesis is related to blood vessel formation. WT1 has been detected in the vasculature of different tumour types^25^,^26^ and its expression in endometrial cancer has been associated with the induction of angiogenesis^27^. In addition, WT1 directly upregulates the expression of the vascular endothelial growth factor (*VEGF*)^28^ as well as regulating its splicing^29^. Many other vascular genes have been identified as potential WT1 targets, including the vascular endothelial cadherin, angiopoietin-2, vascular endothelial growth factor receptors 1 and 2^30^. Most importantly, the endothelial knockout of WT1 has been shown to reduce both tumour angiogenesis and growth *in vivo*^31^, suggesting that this gene is a major regulator of blood vessel formation in cancer.

Finally, an alternative hypothesis relies on the fact that *WT1* is a key regulator of the epithelial/mesenchymal balance during development and therefore it may also play a role in the EMT of tumour cells^1^. Besides being recently linked to chemoresistance^32^,^33^, cancer-related EMT has long been associated with the acquisition of a malignant phenotype by the epithelial tumour cells: features of EMT have been described in breast^34^ and colorectal cancer^35^, mainly at the invasive front of the tumour. This suggests that the EMT may generate migratory cells which leave the primary site, invade the blood vessels and potentially metastasise. This theory on EMT contribution to tumour progression is supported by the fact that many developmental EMT drivers, including SNAIL, SLUG, TWIST and ZEB1, are aberrantly expressed in cancer and significantly correlate with relapse and poor clinical outcomes^36^,^37^,^38^. Importantly, WT1 has been shown to regulate the EMT which occurs in the developing epicardium^2^ as well as the MET which is required for nephrogenesis^3^ and its transcriptional targets include *SNAIL, SLUG* and *TGF-β*^2^,^39^,^40^.

In the present work, we have studied the relationship between WT1 expression and the overall epithelial/mesenchymal balance of breast cancer using *in vitro* cell lines, clinical samples and publicly available gene expression datasets in order to better investigate why WT1 is usually associated with poor prognosis.

## Results

### Different *WT1* isoforms are observed in breast cancer and their expression is higher in the ER-positive subtype

Several groups have described *WT1* expression in breast cancer^5^,^6^,^7^,^8^,^9^,^10^,^11^, however the data is inconsistent and the frequency of *WT1*-positive tumours varies from 23%^6^ to 87%^7^. Such discrepancies may be due to different detection techniques or antibody specificity^11^, but may also be related to differences in the molecular subtype of the tumours or to different *WT1* isoforms.

In order to overcome these limitations, we have performed a more detailed and comprehensive analysis of *WT1* expression in breast cancer using different approaches (*in vivo, in vitro* and *in silico*).

**Figure 1:**
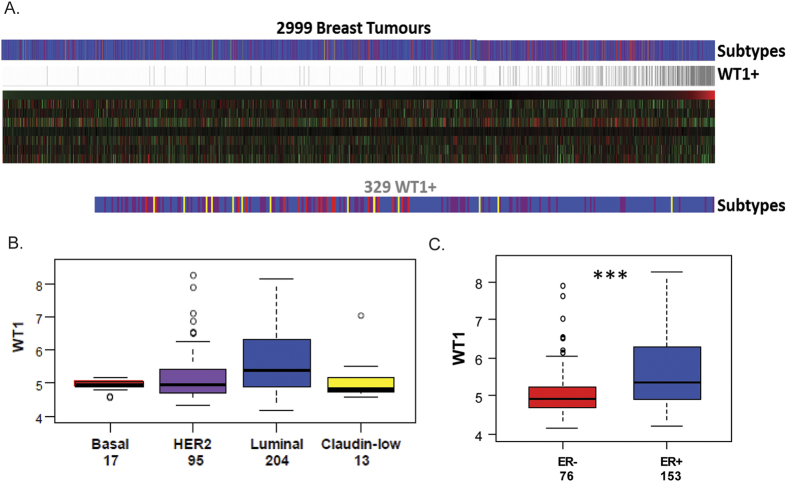
*WT1* expression in human primary breast cancer datasets. (**A**) Gene expression analysis data from 17 integrated datasets (n = 2999 tumours). (**B**) Boxplot of *WT1* expression in the different subtypes, y-axis showing log2 values. (**C**) Boxplots of *WT1* expression in ER-positive (blue) and ER-negative (red) tumours designated by IHC of ER-alpha amongst those tumours with detectable expression of *WT1*, ***p < 0.001.

Firstly, we analysed *WT1* expression in an integrated dataset from 17 studies representing 2999 primary human breast cancers^41^ (Fig. 1A). The transcript could only be reliably detected (considering ‘Present’ detection calls in the Affymetrix data) in 11% of the tumours (329 out of the total 2999) and showed the highest expression level in the luminal subtype (Fig. 1B, Mann Whitney p value of 4.5e-7). Furthermore, restricting the samples to those in which WT1 was detected, *WT1* expression was significantly higher in ER-positive than in ER-negative tumours (Fig. 1C, p = 1.2e-5).

**Figure 2:**
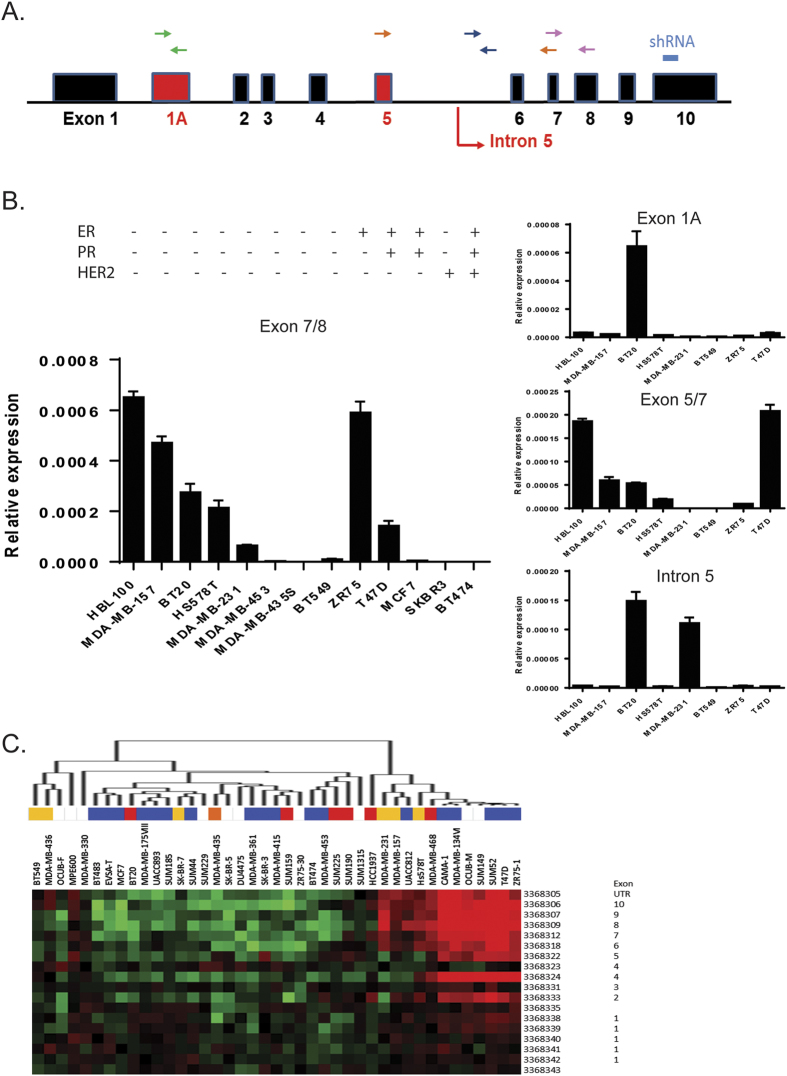
*WT1* expression in breast cancer cell lines. (**A**) Schematic of the *WT1* locus, showing alternative exons/splices in red, the position of the primers for each qRT-PCR assay and the sequence targeted by the shRNA used in the knockdown experiments. (**B**) Quantitative real-time PCR of *WT1* mRNA: data points represent the relative expression for each assay, error bars represent the standard deviation of two biological replicates. (**C**) Heatmap visualization of the relative expression of probes representing each *WT1* exon across a panel of breast cancer cell lines in a published dataset (GSE16732). The different colours indicate the cell line subtype (red = basal, orange = basal B/mesenchymal, purple = HER2 amplified, blue = luminal, yellow = claudin-low).

Secondly, we analysed a locally derived cohort of invasive breast cancer samples (n = 44) using isoform-specific assays: quantitative RT-PCR was first performed using primers targeting exon 7/8 of WT1, in order to amplify all the known isoforms (Fig. 2A, Figure S1 in Additional file 1). Additional assays were then designed to specifically amplify a maternally-imprinted alternative exon 1^42^ and the portion of intron 5 which is transcribed in the truncated variant^43^ (Figure S1 in Additional file 1). To compare the expression levels of the different isoforms between healthy and neoplastic tissue, we also included a sample of human adult mammary gland. This normal tissue sample, a pool from 5 different healthy donors, expressed higher *WT1* levels than any of the tumour samples, but lacked both the exon 1A and intron 5 isoforms.

Lastly, examination of WT1 expression in a panel of thirteen breast cancer cell lines by qPCR demonstrated that seven of the cell lines had detectable levels of WT1, with the highest expression observed in the basal triple-negative lines HBL100 and MDA-MB-157 and in the luminal ER+ line ZR75 (Fig. 2B). While the qPCR for the optional exon 5 mirrors the exon 7/8 results, only BT20 and MDA-MB-231 cells expressed detectable levels of the intron 5 isoform and exon 1A was expressed only by BT20 cells.

To further assess the extraordinary complexity of the *WT1* locus, we also analysed a dataset representing a panel of 41 breast cancer cell lines using Affymetrix exon GeneChips (GSE16732)^44^.

The exon array data was largely in agreement with the qPCR experiments, showing high levels of *WT1* expression for ZR75, MDA-MB-157, HS578T and T47D cell lines (Fig. 2C); moreover, *WT1* expression in MDA-MB-231 cells seemed to be restricted to exons 6 to 10, suggesting that this cell line may only express the truncated intron 5 isoform.

### Knock-down of *WT1* in the MDA-MB-157 line increases the motility, invasiveness and metastatic potential of breast cancer cells

**Figure 3:**
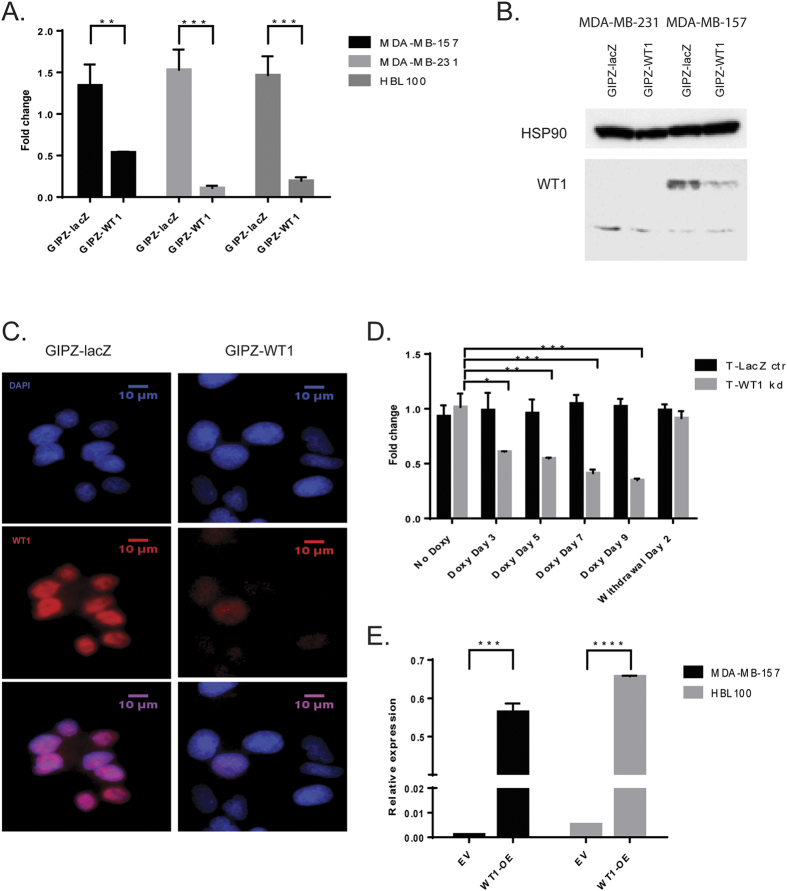
*WT1* knockdown and overexpression is achieved in breast cancer cell lines. (**A**) Quantitative real-time PCR of *WT1* mRNA in MDA-MB-157, MDA-MB-231 and HBL100 cells transduced with pGIPZ-miR: the graph represents fold change of mean expression relative to untransduced cells (given a value of 1); error bars represent the standard deviation of two separate biological replicates (**p < 0.01, ***p < 0.001). (**B**) Immunoblot of whole-protein lysates (40 μg) probed with antibodies to WT1 and HSP90, as a loading control. Only the truncated isoform of WT1 can be observed in MDA-MB-231 cells. Full-length blots are presented in Figure S8 in Additional file 1. (**C**) WT1 immunofluorescence of MDA-MB-157 cells transduced with pGIPZ-miR. (**D**) Quantitative real-time PCR of *WT1* mRNA in MDA-MB-157 cells transduced with pTRIPZ-miR: the cells were cultured on 2 μg/ml doxycycline, the graph represents fold change of mean expression relative to untransduced cells, error bars represent the standard deviation of three biological replicates (*p < 0.05, **p < 0.01, ***p < 0.001). (**E**) Quantitative real-time PCR of *WT1* mRNA in MDA-MB-157 and HBL100 cells transfected with pCI-WT1-OE; data points represent the relative expression, error bars represent the standard deviation of three biological replicates (***p < 0.001, ****p < 0.0001).

An RNAi-based gene knockdown strategy was used to investigate the functional role of *WT1* in breast cancer cells. Unlike previous studies based on transient transfections^45^, we achieved stable *WT1* knockdown in three human breast cancer cell lines (Fig. 3A,B). Both constitutive and inducible lentiviral vectors were used for MDA-MB-157 cells, which showed up to 70% mRNA and protein reduction (Fig. 3A–D).

RNA-seq was used to investigate the consequences of *WT1* loss in the MDA-MB-157 cell line by comparing the transcriptomes of the wildtype and knockdown cells. Differential expression of 427 transcripts (Table S1 in Additional file 2) was observed, including several genes involved in RNA metabolism/biosynthesis, the neuronal gene *SEMA5A* (reported to play a role in cancer progression^46^), and the ncRNA *MALAT1* (a regulator of the metastasis phenotype in lung cancer^47^). ToppFun (http://toppgene.cchmc.org/enrichment.jsp)^48^ was used to functionally annotate the differentially expressed genes and it showed enrichment for genes involved in several migration-related biological processes, from cell adhesion to cell projection morphogenesis/organisation (Table S2 in Additional file 2).

**Figure 4:**
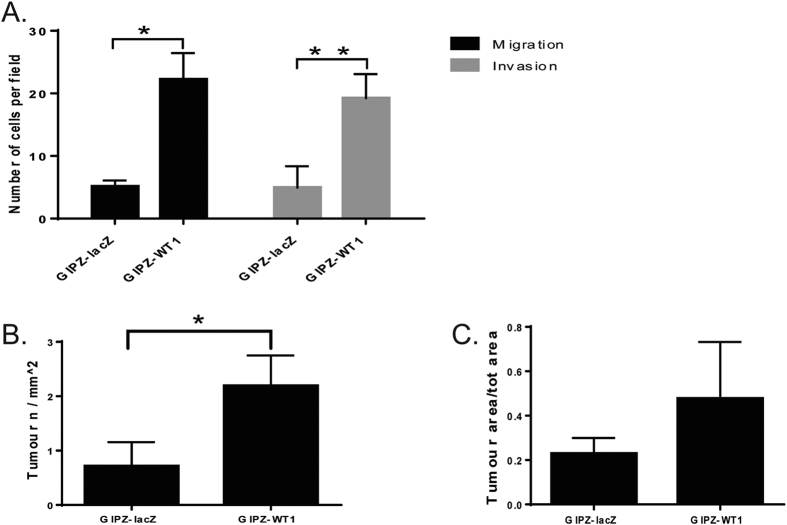
Knockdown of WT1 results in increased motility, invasiveness and metastatic potential of MDA-MB-157 cells. (**A**) Bar diagram representing the mean number of migrating and invading cells from three independent experiments. (**B**,**C**) WT1 knockdown significantly increases the number (**B**) but not the size (**C**) of lung metastases. MDA-MB-157 cells transduced with pGIPZ-miR were injected into nude mice through the tail vein and lung metastases were assessed 8 weeks after injection (n = 4 for each group).

In order to assess if *WT1* knockdown affects the motility of breast cancer cells, we performed migration and invasion assays. The cells in which *WT1* was silenced displayed a statistically significant increase in both migratory capacity and invasiveness when compared to pGIPZ-lacZ controls (p = 0.031 and p = 0.008 respectively) (Fig. 4A).

The knockdown cells also showed significantly increased incidence of lung metastases following injection into the tail vein of nude athymic mice (p = 0.011) (Fig. 4B), although the total tumour area was not significantly different between the two groups (Fig. 4C).

### Knockdown and overexpression of *WT1* disrupts the epithelial-mesenchymal balance of breast cancer cells

Since increased migration is considered a feature of EMT^49^, we determined if the loss of *WT1* also affects the epithelial/mesenchymal balance of breast cancer cells and analysed the RNA levels of several EMT drivers (*SNAI1, SNAI2, ZEB1, ZEB2, TWIST1*), epithelial markers (*CTNNA1, CTNNA2, CTNND1, CDH1, KRT18*) and mesenchymal markers (*VIM, TNC, TGFB1, CDH2, MMP2, MMP9*) through quantitative real-time PCR.

We also generated and analysed overexpression clones from two different lines using the full length cDNA of WT1 (Fig. 3E).

**Figure 5:**
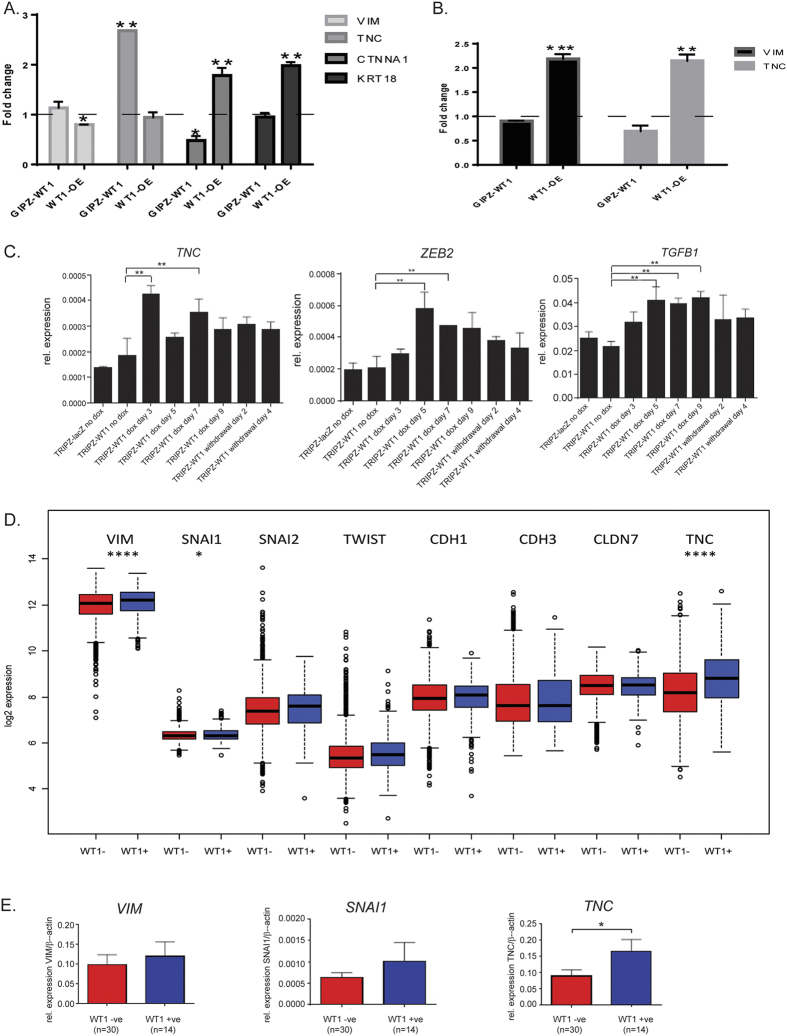
Correlation between WT1 expression and EMT markers *in vitro* and *in vivo*. (**A**) Quantitative real-time PCR of epithelial and mesenchymal markers in MDA-MB-157 cells transduced with pGIPZ-miR or transfected with pCI-WT1-OE: the graph represents fold change of mean expression relative to control cells, error bars represent the standard deviation of three biological replicates (*p < 0.05, **p < 0.01). (**B**) Quantitative real-time PCR of mesenchymal markers in HBL100 cells transduced with pGIPZ-miR or transfected with pCI-WT1-OE: the graph represents fold change of mean expression relative to control cells, error bars represent the standard deviation of three biological replicates (**p < 0.01, ***p < 0.001). (**C**) Quantitative real-time PCR of *TNC, TGFB1* and *ZEB2* mRNA in MDA-MB-157 cells transduced with pTRIPZ-miR: data points represent the relative expression of the gene, error bars represent the standard deviation of three separate biological replicates (**p < 0.01). (**D**) Boxplot of EMT genes expression in the *WT1*-positive and *WT1*-negative samples obtained from 17 integrated datasets (n = 2999 tumours), y-axis showing log2 values. Statistically significant differences were observed only for *VIM, SNAI1* and *TNC*, which showed higher expression in the *WT1*-positive samples. *p < 0.05, ****p < 0.0001. (**E**) Quantitative RT-PCR of EMT genes in clinical samples (n = 44 tumours).

**Figure 6:**
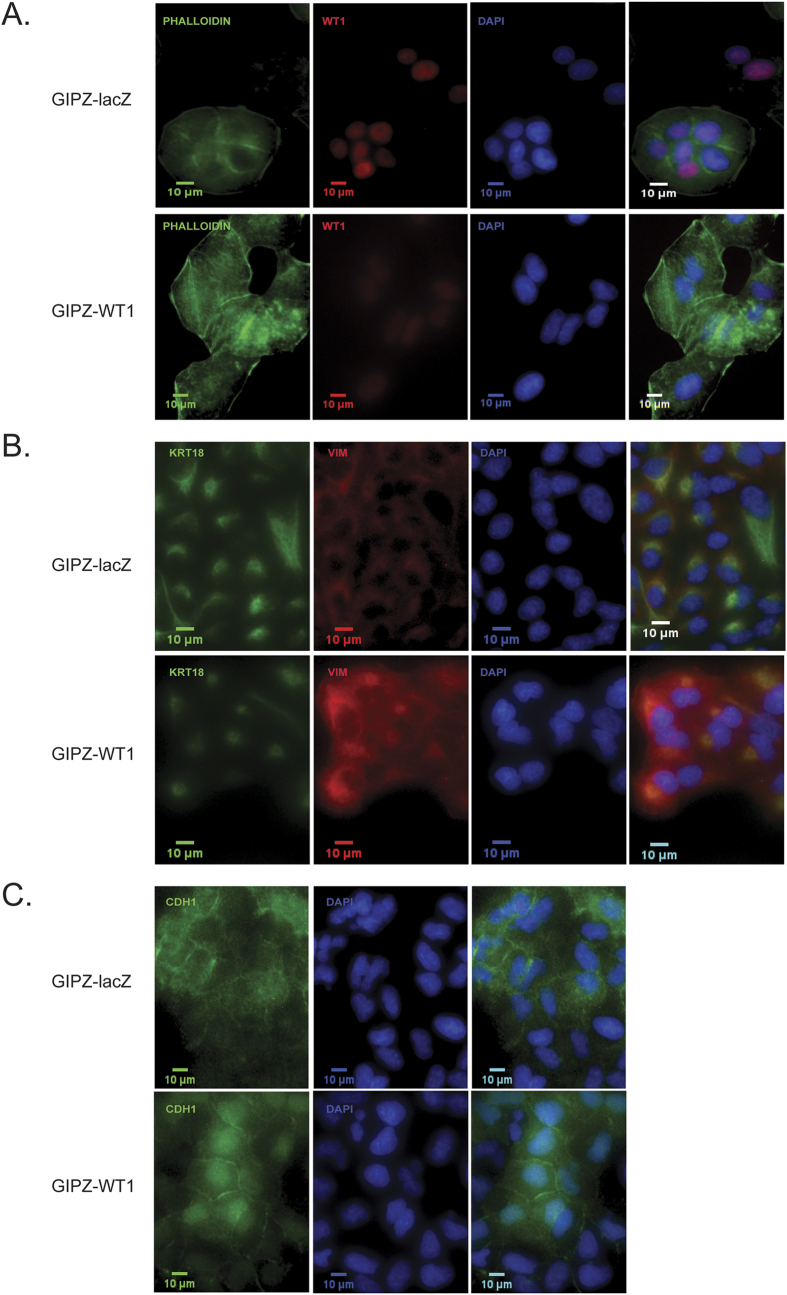
EMT markers immunofluorescence in MDA-MB-157 cells. MDA-MB-157 cells transduced with pGIPZ-miR were stained with phalloidin toxin (**A**), vimentin/cytokeratin18 (**B**) and E-cadherin (**C**) antibodies.

In the mesenchymal line MDA-MB-157, overexpressing *WT1* pushes the cells towards a more epithelial phenotype, with increased expression of *CTNNA1* and *KRT18* as well as downregulation of *VIM* (Fig. 5A). On the other hand, silencing *WT1* exacerbates their mesenchymal characteristics, with significant changes in the expression of EMT markers (Fig. 5A–C) along with a more elongated and irregular morphology, as shown by phalloidin staining (Fig. 6A). Moreover, even though no significant differences are observed at the mRNA level, the knockdown cells display nuclear accumulation of CDH1 (Fig. 6C), a feature that has been linked to increased tumour invasion^50^.

Some of these results, such us the upregulation of *TNC* upon WT1 loss, were replicated in the other mesenchymal line used in this study, MDA-MB-231 (Figure S3 in Additional file 1). However, completely opposite results were observed in the epithelial line HBL100, where WT1 overexpression led to the up-regulation of mesenchymal markers (Fig. 5B).

In order to understand which scenario more closely represents what happens *in vivo*, we compared the expression of EMT-related transcripts (*VIM, SNAI1, SNAI2, TWIST1, CDH1, CDH3, CLDN7, TNC*) between *WT1*-positive and *WT1*-negative samples from the integrated dataset described in Fig. 1A: statistically significant differences were only observed for the mesenchymal genes *VIM, SNAI1* and *TNC*, which showed higher expression in the *WT1*-positive samples (p-value < 0.001, <0.05, <0.001 respectively) (Fig. 5D, Figure S4 in Additional file 1).

The results were further validated with the locally derived cohort previously described: the patient samples were first sorted according to their levels of *WT1* (Figure S1 in Additional file 1), then qPCR was used to assess the expression of the mesenchymal markers *VIM, SNAI1* and *TNC* (Fig. 5E). The *WT1*-postive tumours had higher expression of all three markers, even though statistical significance was only achieved for *TNC*.

### Down-regulation of *WT1* does not result in changes in proliferation, apoptosis or cancer stem cell characteristics

Transient silencing of *WT1* has been previously shown to inhibit proliferation and induce apoptosis in cultures originated from leukemic cells and several solid tumours^16^,^17^,^18^,^45^, therefore we also performed apoptosis and cell cycle analysis. However, our knockdown cells did not show any significant change in either of these (Figures S5–6 in Additional file 1), suggesting that, at least for breast cancer cells with stable WT1 knockdown, the main phenotype is the disruption of the epithelial/mesenchymal balance, rather than apoptosis or cell cycle effects.

Since the EMT process has been linked to the acquisition of cancer stem-cell properties (e.g., an increased ability to form spheres in colony-forming assays or the expression of stem-cell markers)^51^, we also analysed stem-cell characteristics in the *WT1* knockdown cells.

**Figure 7:**
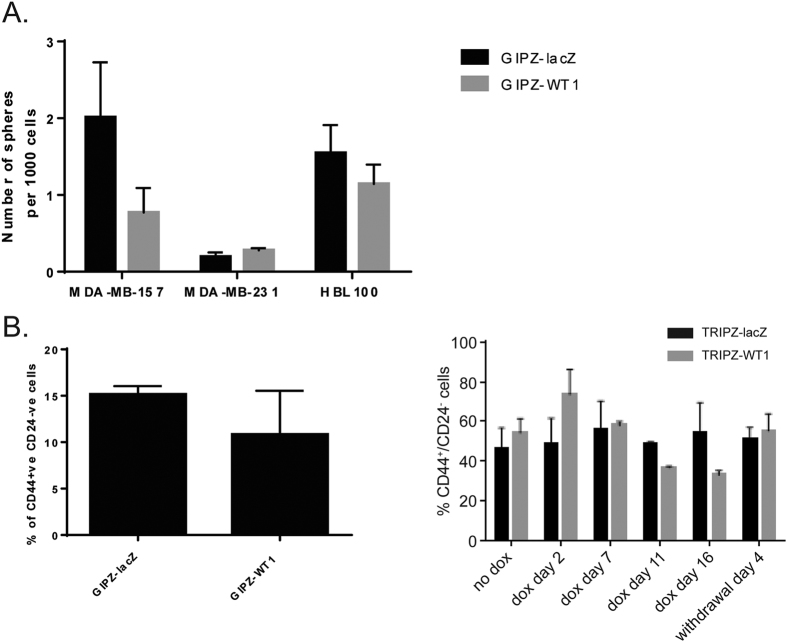
Knockdown of WT1 does not affect CSCs percentage or colony forming ability. (**A**) Colony forming assay of MDA-MB-157, MDA-MB-231 and HBL100 cells with constitutive WT1 knockdown suspended in semi-solid medium. Data points represent the average number of spheres formed per well, error bars represent the SEM of four biological replicates. (**B**) FACS analysis of CD24 and CD44 expression in MDA-MB-157 cells transduced with pGIPZ-miR and pTRIZ-miR. Bar graphs represent the average percentage of CD44+/CD24− cells in the different clones, error bars represent the SEM of three biological replicates.

No statistically significant difference in mammosphere formation was observed in any of the three lines used in this study (Fig. 7A). The cancer stem cell content of the knockdown cells was analysed by quantifying the sub-population of CD44+/CD24− cells, which has been reported to be highly enriched in CSCs^51^. In the MDA-MB-157 line the percentage of CD44+/CD24− cells did not change significantly upon WT1 loss (Fig. 7B), suggesting that down-regulation of WT1 does not affect the ‘stemness’ of breast cancer cells.

### *WT1* expression correlates with *CYP3A4* levels and is associated with poorer response to taxane treatment

**Figure 8:**
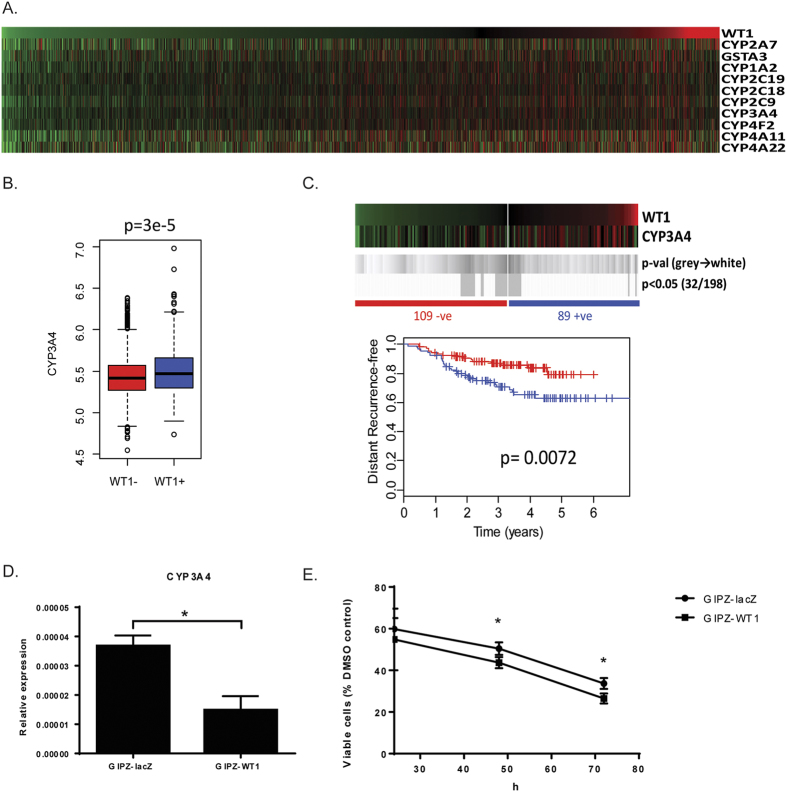
*WT1* expression in breast cancer correlates with cytochrome P450 family members and poor response to taxane treatment. (**A**) Heatmap showing ranked expression of WT1 alongside cytochrome P450 genes obtained from 17 integrated published microarray datasets (n = 2999 tumours). (**B**) Boxplot of CYP3A4 expression in the WT1-positive and WT1-negative samples. (**C**) Kaplan Meier survival analysis of 198 breast cancer patients treated with taxane-anthracycline chemotherapy (Dataset GSE25065) demonstrates that WT1 expression is associated with worse outcomes. Heatmaps show log2 mean-centered expression values, red = high, green = low. Shades of grey to white indicate p-values of log-rank (Mantel-Cox) tests at all possible cut-points are shown in grey. Vertical bars on survival curves indicate censored cases. (**D**) Quantitative real-time PCR of CYP3A4 mRNA in MDA-MB-157 cells transduced with pGIPZ-miR: data points represent the relative expression of the gene, error bars represent the standard deviation of two separate biological replicates (*p < 0.05). (**E**) Cytotoxycity of docetaxel is increased in MDA-MB-157 cells transduced with pGIPZ-miR. Mean and standard deviation of three independent experiments are plotted (*p < 0.05).

In order to identify differentially expressed genes between *WT1*-positive and *WT1*-negative breast tumours, significance analysis of microarray (SAM) was performed using the publicly available datasets previously described^41^. Among the 500 most significantly differentially expressed transcripts (Table S3 in Additional file 2) there are several different types of metalloproteases and many genes belonging to the cytochrome P450 family (CYP), all of which are significantly (p < 0.0001) positively correlated with WT1 (Fig. 8A).

These oxidising enzymes play an important and complex role in the metabolism of anti-cancer agents: they participate in the biological activation of many pro-drugs, but also to the inactivation of some cytotoxic compounds, two processes that occur predominantly, but not exclusively, in the liver. Not surprisingly, polymorphisms and different expression levels of the CYP genes have been shown to influence the outcome of chemotherapeutic treatments^52^.

The substrates of one of these up-regulated genes, *CYP3A4*, include drugs routinely used in the management of breast cancer such as tamoxifen, docetaxel, paclitaxel, cyclophosphamide and doxorubicin^52^. More specifically, CYP3A4 activity in the liver is responsible for the activation of tamoxifen and cyclophosphamide, whereas it transforms docetaxel into inactive hydroxylated derivatives; interestingly, high *CYP3A4* levels in breast cancer biopsies can predict a poor therapeutic response to docetaxel, which suggests that also the enzyme within the tumour is involved in the inactivation of this drug^53^.

Given this association between *WT1* and *CYP3A4* expression in breast cancer (Fig. 8B), we decided to investigate whether the tumour levels of *WT1* can be used to predict the response of breast cancer patients to docetaxel. To do so, we analysed a publicly available gene expression dataset (GSE25065) of 198 HER2-negative breast cancer cases treated with taxane-anthracycline chemotherapy pre-operatively and endocrine therapy if ER-positive^54^. As shown in Fig. 8C, *WT1* expression is significantly associated with poorer response and survival, with the 5-year recurrence-free percentage in the WT1-positive patients being 20% lower than in the WT1-negative (p value = 0.0072). To further validate these results, *CYP3A4* expression levels were analysed in the MDA-MB-157 line and showed significant down-regulation upon *WT1* loss (p = 0.035) (Fig. 8D); the WT1 knockdown cells also displayed increased sensitivity to docetaxel if compared to GIPZ-lacZ control (Fig. 8E).

## Discussion

Despite extensive studies, the role of *WT1* in breast cancer and its significance in terms of prognosis remain unclear, with conflicting results being reported in the literature.

Since this malignancy is a very heterogeneous disease and the WT1 locus is extremely complex, we started our study with a comprehensive assessment of *WT1* expression in the different tumour subtypes followed by an isoform specific expression analysis.

Our work shows that *WT1* expression in breast cancer occurs at low frequency (10–30%), and it is lower than in the healthy mammary gland, which is in agreement with some of the earlier studies on the topic^5^,^6^. Also, *WT1* expression is significantly higher in ER-positive (luminal) than in ER-negative (basal) tumours. This finding may be of particular interest, given that WT1 has been shown to both interact with ERα and modulate its expression *in vitro*^55^.

In terms of isoform expression pattern, all our data corroborate the hypothesis that the truncated transcript starting from intron5 is a tumour-specific isoform: this short *WT1* variant was first detected in the blood of patients affected by acute leukaemia, then in breast and pancreatic cancer cell lines^43^,^56^ and we have now shown that it is expressed in human breast cancer biopsies, but is absent in the healthy mammary gland.

Both our *in vitro* and *in silico* experiments also seem to demonstrate that the truncated intron5 is the only *WT1* isoform expressed in MDA-MB-231 cells; if confirmed, any assay targeted against exon1/5 would give a negative result, which may explain why the reports on *WT1* expression in this cell line are so contradictory^7^,^24^,^45^,^57^,^58^.

The last isoform we analysed, derived from a maternally imprinted alternative exon 1^42^, has been detected in few human breast cancer samples and in only one cell line, suggesting that it is unlikely to play a major role in breast tumorigenesis.

WT1 expression in invasive ductal carcinoma has been associated with both improved^14^ and worse^8^ outcomes, while a third study, specifically on triple negative breast cancers^15^ has shown that high WT1 levels correlate with poor survival. *WT1* expression in other adult tumours is usually associated with higher histological grade as well as worse prognosis^59^,^60^ and several hypotheses have been formulated to explain this association, such as altered proliferation/apoptosis^16^,^17^,^18^,^19^, increased angiogenesis^25^,^26^,^27^ and induction of cancer-EMT^1^.

In this study, we focused our analysis on the relationship between WT1 expression and the overall epithelial/mesenchymal balance of breast cancer cells by developing the first stable *WT1* knockdown in human breast cancer cells and extensively studying the effects of *WT1* silencing as well as overexpression.

When we perturb the expression levels of WT1, either by knocking it down or over-expressing it, the breast cancer cells show a disruption of their epithelial-mesenchymal balance, which seems to be dependent on their baseline characteristics. In particular, in mesenchymal-leaning lines such as MDA-MB-157 and MDA-MB-231, the loss of WT1 pushes them towards an even less epithelial phenotype, with increased motility, invasiveness and metastatic potential (as shown by the tail vein injection experiment). The opposite holds true for the epithelial line HBL100, where it is WT1 over-expression that leads to a more mesenchymal state. Such a dichotomous role is not surprising for WT1, which is known to regulate both EMT and MET in development^2^,^3^.

In terms of gene expression, the most significant changes are observed for the epithelial marker *CTNNA1* (a catenin that maintains normal cell adhesion by forming a complex that anchors E-cadherin to the cell membrane and if disrupted can lead to tumour invasion) and the mesenchymal marker *TNC* (an extracellular matrix glycoprotein which is usually found at the invasive front of the tumour^61^ and has been shown to induce EMT-like changes and faster migration in breast cancer cells^62^). Interestingly, *VEGF*, which is an established *WT1* target^28^ is likely upstream of *TNC*^63^.

Overall, perturbing the expression levels of *WT1* affects the epithelial/mesenchymal balance of cancer cells, but does not cause any significant change in cancer stem-cell properties, cell proliferation or apoptosis.

This finding is apparently in contrast with some previous experiments on leukemic and solid tumours cell lines, where the transient silencing of *WT1* led to growth arrest and apoptosis^16^,^17^,^18^,^45^. However, the activation of an EMT program has been shown to protect from pro-apoptotic signals^64^: this could explain the discrepancy for MDA-MB-157 and MDA-MB-231 cells, which increase their mesenchymal features upon WT1 loss.

The *in vitro* experiments were then corroborated by the analysis of publicly available gene expression datasets of breast cancer cell lines and clinical samples: overall, the results are consistent with what observed in the HBL100 cell line and suggest that *WT1* expression is usually associated with a more mesenchymal phenotype.

The gene expression analysis also revealed that the *WT1*-positive tumours exhibit poorer response to taxane treatment, which may be linked to their increased levels of *CYP3A4*, an oxidising enzyme which transforms docetaxel into inactive hydroxylated derivatives^65^. Importantly, *CYP3A4*, whose expression levels correlate with *WT1* both *in vivo* and *in vitro*, is activated by VDR^66^, a known WT1 target^67^.

As a whole, this study has shown that WT1 can disrupt the epithelial-mesenchymal balance of breast cancer lines in opposite ways, depending on the baseline characteristics of the cells.

In breast tumours however, the *WT1*-expressing tumours are mainly associated with a mesenchymal phenotype and high levels of CYP3A4: therefore, their cells not only respond poorly to taxane, but they are also more likely to invade the blood vessels and metastasise.

In conclusion, we demonstrate that cancer-related EMT and poor chemotherapy response are likely the main factors that explain why WT1 expression in breast cancer is usually associated with poor prognosis.

## Methods

### Cell culture

The human breast cancer cell lines MDA-MB-157, MDA-MB-231, MDA-MB-453, MDA-MB-435S, HBL100, MCF7, BT20, SKBR3, T47D, HS578T and ZR75 were maintained in DMEM medium (Sigma); BT474 and BT549 cell lines in RPMI medium (Sigma). Both media were supplemented with 10% fetal bovine serum (FBS), 2 mM L-glutamine, 100 units/mL penicillin and 100 mg/ml streptomycin. The human embryonic kidney cell line SODK3 used for the lentiviral packaging was maintained in DMEM-HIGH (Sigma) supplemented with 10% FBS, 2 mM L-glutamine, 100 units/mL penicillin, 100 mg/ml streptomycin, 1 μg/ml puromycin, 600 μg/ml neomycin and 1 μg/ml doxycycline. All lines were kept at standard cell culture conditions (37 °C, 5% CO2 in humidified incubator). Tet System Approved FBS (Clontech) was used in the culture media of TRIPZ-transduced cells, to ensure that the inducible regulation of the system was not altered by the presence tetracycline-derived contaminants.

### Clinical samples

The use of tissue from invasive breast cancer patients treated at the Edinburgh Breast Unit at the Western General Hospital was approved by the Lothian Research Ethics Committee (08/S1101/41). This study was carried out in accordance with the approved guidelines and written informed consent was obtained from all subjects. Multiple core biopsies were harvested from consenting patients at the time of curative surgical resection for invasive breast cancer. Macroscopically distinct fat was trimmed and discarded. Tumour samples were then stored at −80 °C until use.

### shRNA-based stable WT1 knockdown and transient WT1 overexpression

The lentiviral GIPZ-shRNAmir-GFP and TRIPZ-shRNAmir-RFP vectors (Thermo Scientific Open Biosystems) were used as backbones for the cloning of shRNAs targeting the human *WT1* transcript and a lacZ control (Figure S7 in Additional file 1, Table S4 in Additional file 2).

The final constructs were transduced using a trans-lentiviral packaging system developed in the human embryonic kidney cell line SODK3^68^, which expresses GFP and all trans-lentivirus packaging proteins from a Tet-Off system. To activate the packaging system, the cells were grown for 3 days without doxycycline and FACS sorted for GFP expression; the brightest 15% was plated on a 0.001% poly-L-Lysine coated plate and grown overnight without doxycycline. The following day, the SODK3 cells were transfected with the lentiviral vectors using FuGene6 (Roche) as per manufacturer’s instructions; the medium, containing 5 mM sodium butyrate (Sigma) but no doxycycline, was changed at 12 and 36 hours post-transfection. 72 hours after the last medium change, the virus-containing supernatants were harvested and centrifuged for 20 minutes at 4 °C to pellet cell debris. Standard procedures were used to transduce MDA-MB-157, MDA-MB-231 and HBL100 cells, which were maintained on selective media (1 μg/ml puromycin) from day 2 post-transduction.

Full-length WT1 cDNA was amplified and cloned into the pCI mammalian expression vector using In-Fusion (Clontech). Cells were transfected with pCI-WT1-OE or the empty vector (Figure S7 in Additional file 1) and harvested after 30 h.

### Cytotoxicity assay

Cells were plated onto 96-well plates (5 × 10^3^ cells/well) and after 24 hours the culture medium was replaced by fresh medium containing 40 nM docetaxel or DMSO. The cells were incubated for another 24, 48 and 72 h, then washed with PBS, fixed in cold methanol and stained with 0.5% crystal violet for 30 minutes. After adding 100 μl Sorenson buffer/well, absorbance was measured as a read-out of viable cells.

### Animal experiments

Lung colonization experiments were performed in compliance with the UK Animals (Scientific Procedures) Act 1986, under Project Licence PPL 60/3788 approved by the UK Home Office. 1 × 10^6^ cells suspended in 100 μl sterile PBS were directly injected into the lateral tail vein of 4-week-old female athymic nude mice. Lung metastases were identified by histological staining of paraffin sections with hematoxylin and eosin. The slides were scanned with a Hamamatsu nanozoomer slide scanner (Hamamatsu, UK), and then examined by two boarded pathologists -blinded to the experimental groups- for the presence of metastases using Hamamatsu NDP2 view software (Hamamatsu, UK). One representative section was chosen for each pulmonary lobe available for examination and measured. The measurements included total number of metastatic deposits, and the relative area of metastasis, expressed as total metastatic area/total lobe area for each sample.

### Colony forming assay

Sphere formation was induced by culturing the cancer cells in suspension in serum-free medium (DMEM-F12 + GlutaMAX-I, Life Technologies) supplemented with B27 (1:50, Life Technologies), 20 ng/mL EGF (R&D), 0.4% bovine serum albumin (Sigma), 4 μg/mL insulin (Sigma). To quantify colony formation, cells were plated at a density of 1,000 cells per well in 96-well plates coated with poly(2-hydroxyethyl metacrylate) to prevent cell attachment to the surface (Sigma); cells were diluted in supplemented serum-free media containing 1% methylcellulose (Sigma) and the number of colonies >75 μm in diameter was counted after 11 days^38^.

### Immunofluorescence microscopy

Cells were fixed for 15 minutes in 4% paraformaldehyde, rinsed with PBS and permeabilised for 10 minutes in 0.1% Triton X-100. Nonspecific immunoglobulin binding was blocked with a 30 minute incubation in 3% bovine serum albumin +0.1% Triton X-100. Primary antibodies (E-cadherin Cell Signalling 24E10, Vimentin Cell Signalling 5741P, Wt1 Abcam 89901, Cytokeratin18 Santa Cruz 31700) were diluted 1:100 in 1% bovine serum albumin +0.1% Triton X-100. After overnight incubation at 4 °C, cells were rinsed with PBS, then incubated for 1 h at room temperature with secondary antibodies (1:500 Alexa Fluor Invitrogen) or Phalloidin (Invitrogen A12379). Vectashield with DAPI (Vector Laboratories) was used to counterstain the nuclei and mount the slides.

### Migration and invasion assays

Migration assays were performed on 24 well plates containing 8 μm PET culture inserts (Fisher Scientific). For each experiment, 5 × 10^4^ cells resuspended in serum-free media were applied to the upper chamber while the lower chamber was filled with 300 μl medium with 10% FBS. Cells were then incubated under normal conditions for 3.5 hours.

The invasion assay was similar to the migration assay, except that the inserts were evenly coated with 1 mg/ml Matrigel; 2.5 × 10^4^ cells were added to the upper chamber and incubated for 24 h. For both assays, cells were fixed with methanol for 10 min and stained with 0.5% crystal violet for 30 min. After removing the cells on the upper side of the filters with cotton-tipped swabs, the cells on the underside of the filters were counted under a X20 objective lens in five randomly chosen fields.

### Cancer stem cell markers, apoptosis and cell cycle analysis

For the analysis of cancer stem cell markers, 1 × 10^6^ cells were resuspended in 100 μl PBS/5% FBS and incubated in the antibody dilution shown in Table S5 in Additional File 2. The GIPZ-transduced cells were incubated with CD44-APC and CD24-PE antibodies, the TRIPZ-transduced cells with CD44-APC and CD24-FITC. After a 15 minute incubation on ice, the cells were washed twice in PBS, resuspended in 100 μl PBS/5% FBS and analysed. The Annexin V Apoptosis Detection Kit APC (eBioscience) was used according to the manufacturer’s instructions: cells were washed once and resuspended at a concentration of 1 × 10^6^ cells/ml in the binding buffer provided with the kit; 5 μl of the APC-conjugated antibody were added to 100 μl of cell suspension and incubated for 15 minutes at room temperature. After one wash, cells were resupended in 200 μl of binding buffer and 1 μg/ml DAPI (Sigma) was added immediately before the analysis. For cell cycle analysis, cells were trypsinised and resuspended in 1.2 ml PBS; 3 ml ice cold 95% ethanol were then added dropwise while vortexing, to obtain a final 70% ethanol solution where the cells were incubated for 30 minutes.

All experiments were performed on a FACSAriaII Flow Cytometer (Becton Dickinson), data were analysed using FlowJo software.

### RNA extraction, quantitative real-time PCR and RNA sequencing

Total RNA was extracted using RNeasy Mini Kit (Qiagen) with on-column DNAse digestion as per manufacturer’s instructions; cDNA was synthesized from 2 μg total RNA using AMV reverse transcriptase (Roche) and oligo d(T) priming. All the quantitative Real Time PCR experiments were performed in triplicate on the Roche Lightcycler 480 system using Universal Probe Library probes and primers designed with the Universal Probe Library Assay Design Centre (Table S6 in Additional file 1). Relative quantification of gene expression was performed by calculating ΔCt where ΔCt = CtX – CtACTB. The fold change in gene expression between two samples was then determined by calculating 2−ΔΔCt. For RNA sequencing, total RNA was isolated as previously described and sent to GATC Biotech (Germany) for library preparation and sequencing on the Illumina HiSeq 2000 platform. The raw data were analysed using Galaxy (https://usegalaxy.org/) and have been deposited in NCBI’s Gene Expression Omnibus^69^, where they are accessible through GEO Series accession number GSE93636.

An RNA sample of human adult mammary gland was purchased from AMSBIO and retrotranscribed; the specimen consisted of a pool from 5 healthy donors of different age (36, 57, 76, 78 and 83 years old).

### Gene expression analysis

Raw data (.cel) files from seventeen Affymetrix U133A/plus2 primary breast tumour gene expression datasets were downloaded from NCBI GEO (GSE12276, GSE21653, GSE3744, GSE5460, GSE2109, GSE1561, GSE17907, GSE2990, GSE7390, GSE11121, GSE16716, GSE2034, GSE1456, GSE6532, GSE3494) or the caBIG (geral-00143) repositories, normalised, batch-corrected and analysed as previously described^41^. The molecular subtypes were assigned based upon the highest correlation to the Prat *et al*.^70^ centroids applied to each dataset separately. The Affymetrix U133A primary breast tumour gene expression dataset GSE25065^54^ was used to compare the response to taxane-anthracycline chemotherapy in WT1-positive and WT1-negative tumours.

### Immunoblot analysis

Immunoblotting was performed according to standard procedures using antibodies against WT1 (Novus Biologicals NBP1-40787, diluted 1/2000) and HSP90 (BD Bioscience 610419, diluted 1/5000).

## Additional Information

**How to cite this article:** Artibani, M. *et al*. *WT1* expression in breast cancer disrupts the epithelial/mesenchymal balance of tumour cells and correlates with the metabolic response to docetaxel. *Sci. Rep.* **7**, 45255; doi: 10.1038/srep45255 (2017).

**Publisher's note:** Springer Nature remains neutral with regard to jurisdictional claims in published maps and institutional affiliations.

## Supplementary Material

Supplementary Information

Supplementary Dataset
