# Supplementary material for: WT1 expression in breast cancer disrupts the epithelial/mesenchymal balance of tumour cells and correlates with the metabolic response to docetaxel
Source: Sci Rep. 2017 Mar 27;7:45255. doi: 10.1038/srep45255 (PMC5366898; doi:10.1038/srep45255)
Supplement: Supplementary Information [file srep45255-s1.doc]

*WT1* expression in breast cancer disrupts the epithelial/mesenchymal balance of tumour cells and correlates with the metabolic response to docetaxel.

Mara Artibani, Andrew H. Sims, Joan Slight, Stuart Aitken, Anna Thornburn, Morwenna Muir, Valerie G. Brunton, Jorge Del-Pozo, Linda R. Morrison, Elad Katz, Nicholas D. Hastie and Peter Hohenstein.

**Additional file 1**

**Figure S1. Assessing *WT1* expression in the clinical samples.**

Quantitative RT-PCR for *WT1* mRNA expression (exon 7/8, exon 1A, intron 5); data points represent the relative expression, error bars represent the standard deviation of three technical replicates. Samples with a relative expression > 0.001 were considered positive.

**Figure S2. Immunofluorescence of untreated MDA-MB-157 cells.**


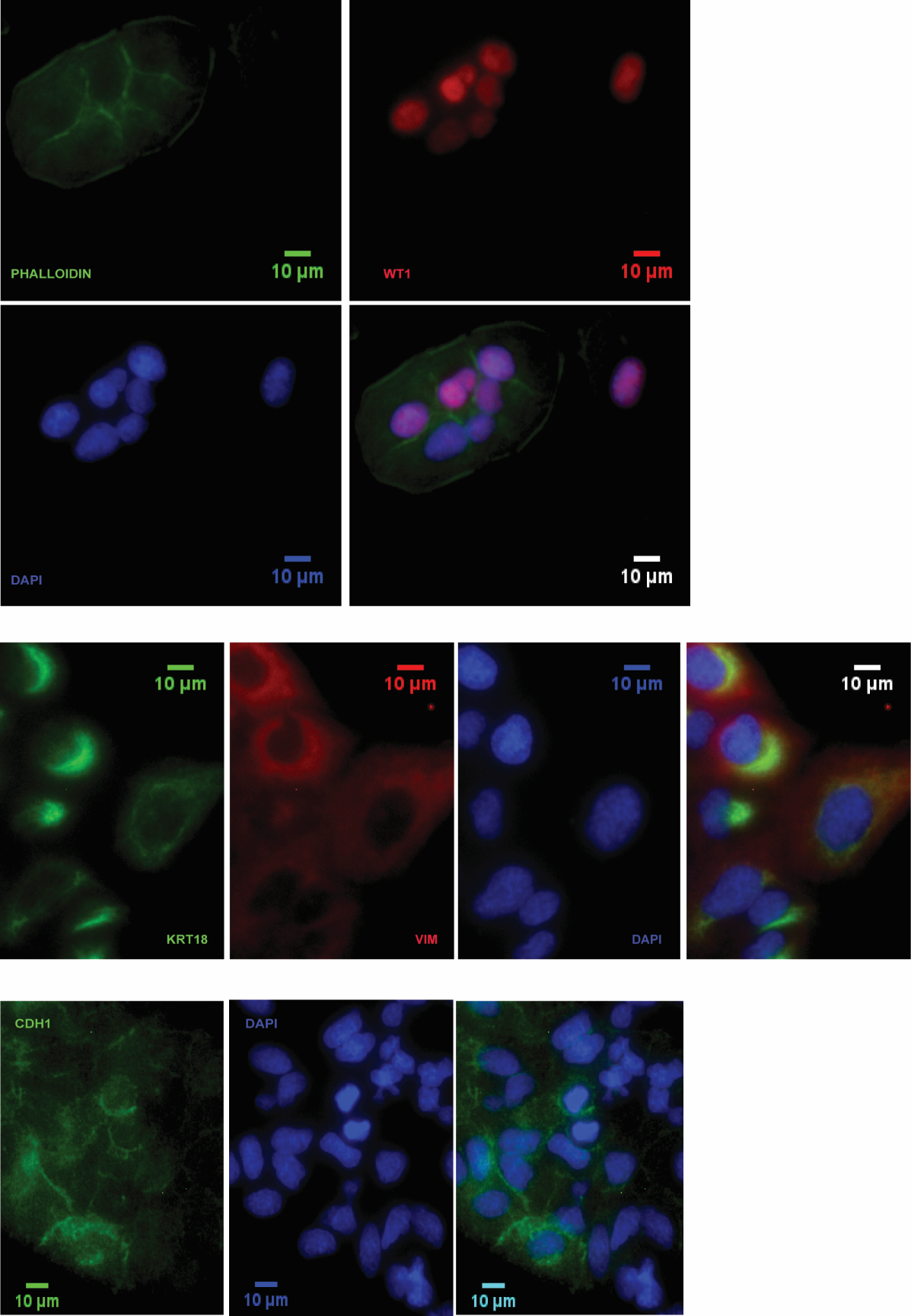


**Figure S3. Knockdown of WT1 in MDA-MB-231 cells induces TNC upregulation.**

Quantitative RT-PCR for *TNC* mRNA expression; data points represent the relative expression, error bars represent the standard deviation of two technical replicates (** p<0.01).

**Figure S4. Expression of *WT1* and EMT markers in human primary breast cancer datasets.**

**
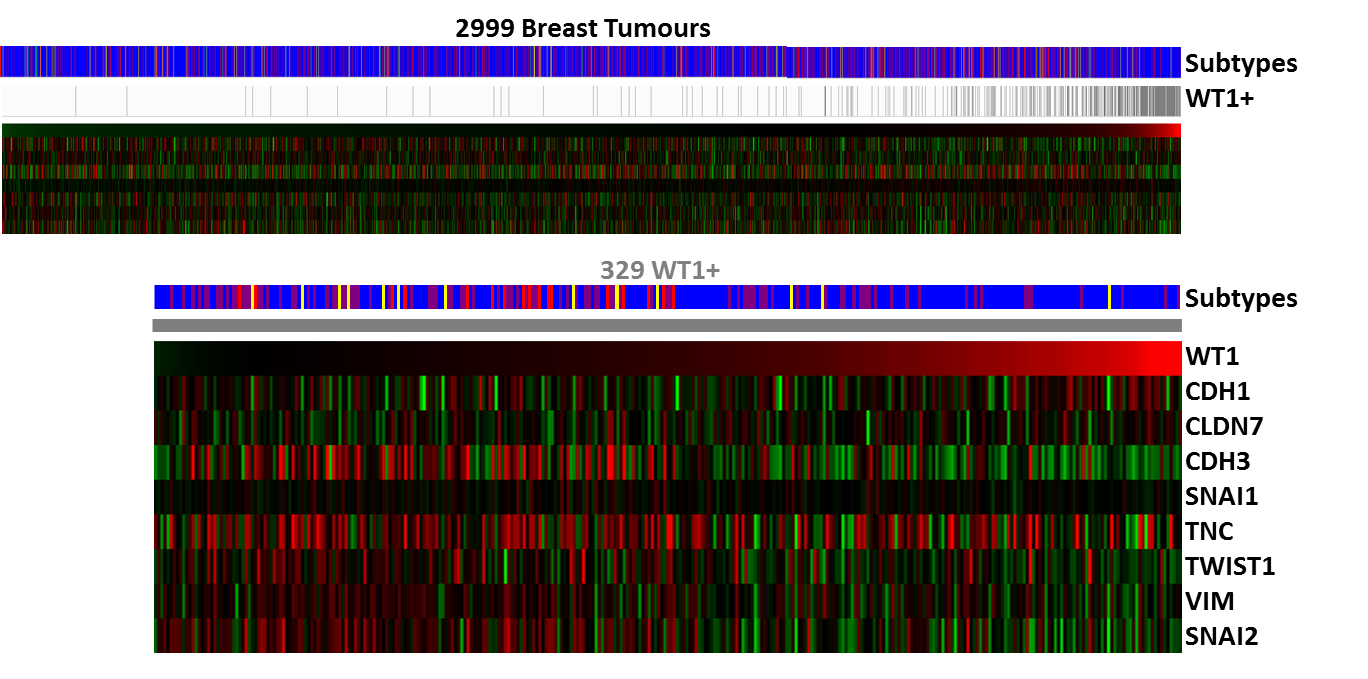
**

**Figure S5. Knockdown of WT1 does not affect apoptosis.**

FACS analysis of Annexin V-APC in MDA-MB-157, MDA-MB-231 and HBL100 cells transduced with constitutive (green) or inducible (blue) constructs. The results are presented as mean values ± SEM of three independent experiments.


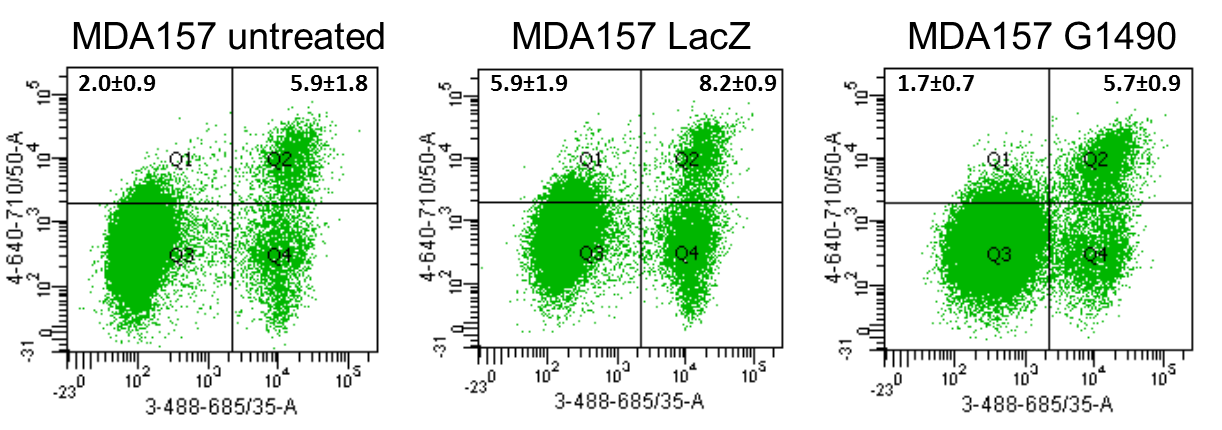


ANNEXIN V


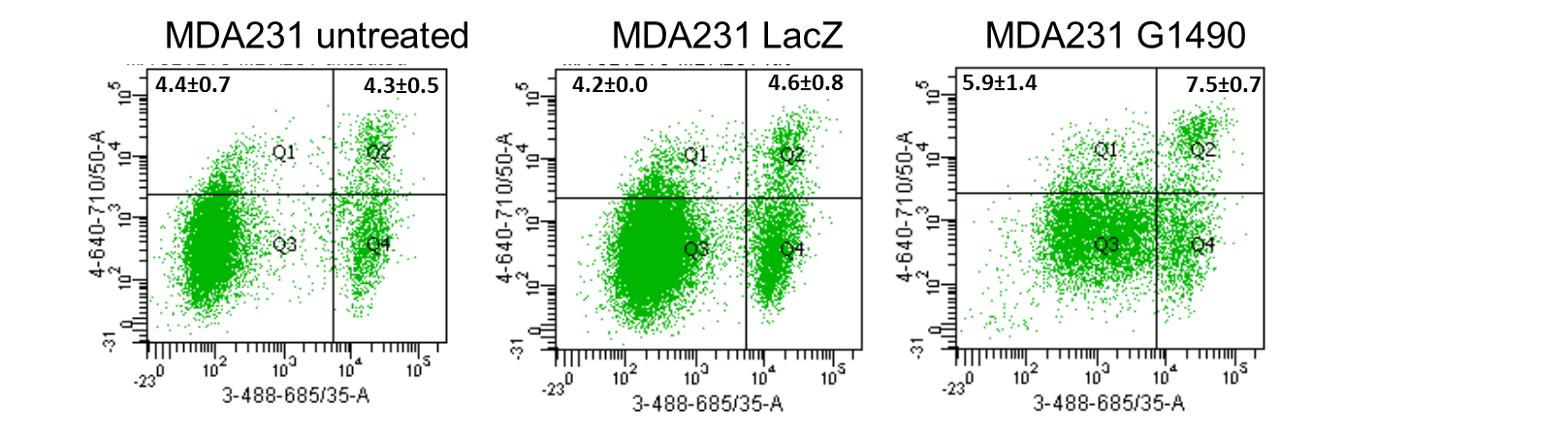


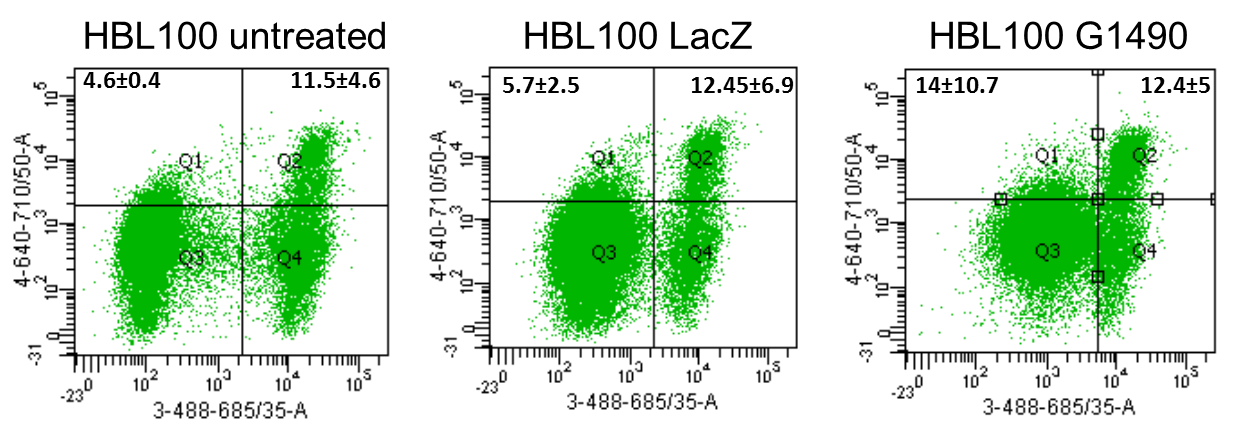


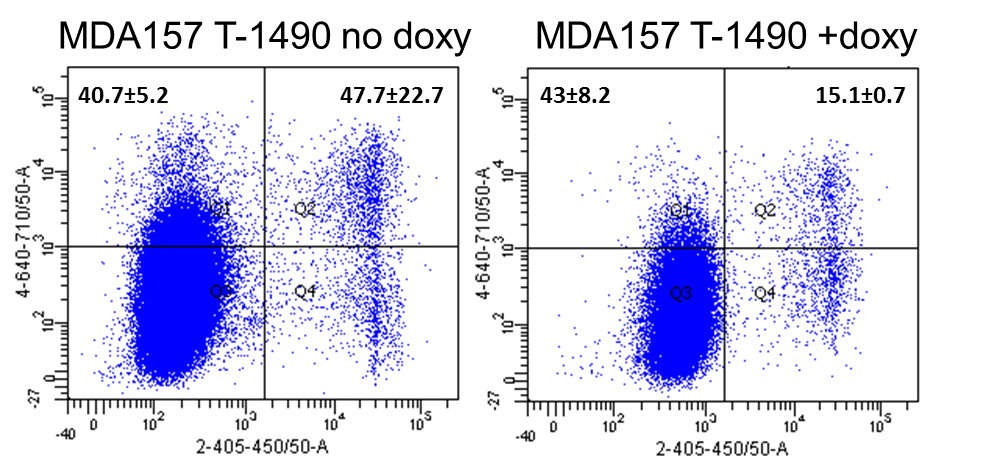


DAPI

**Figure S6. Knockdown of WT1 does not affect cell proliferation.**

FACS analysis of the cell cycle using DAPI staining in MDA-MB-157, MDA-MB-231 and HBL100 cells. The results are presented as mean values ± SEM of three independent experiments.

**Figure S7. Maps of the constructs used in this study: pGIPZ-miR, pTRIPZ-miR and pCI-WT1-OE.**


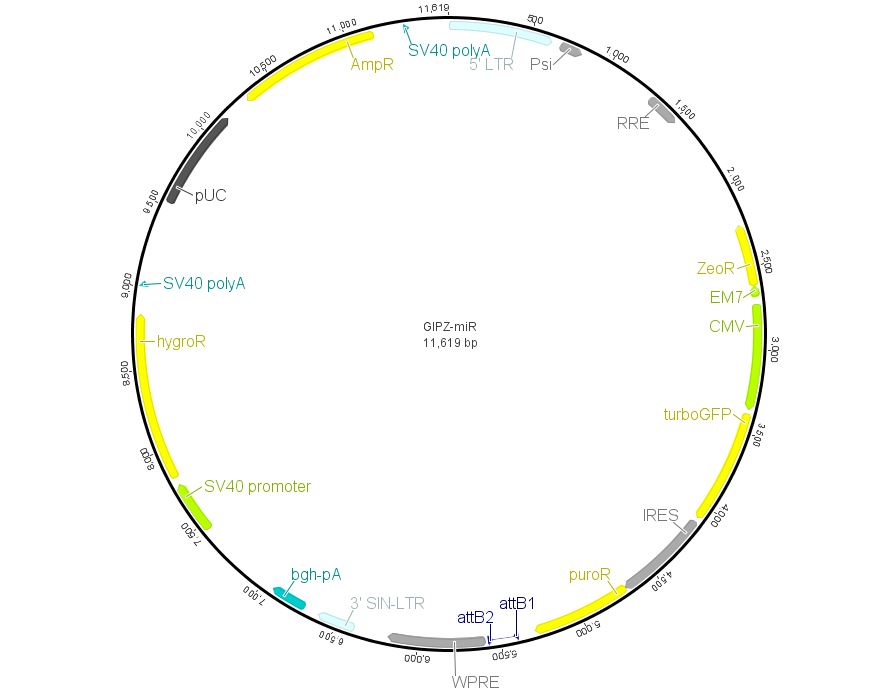


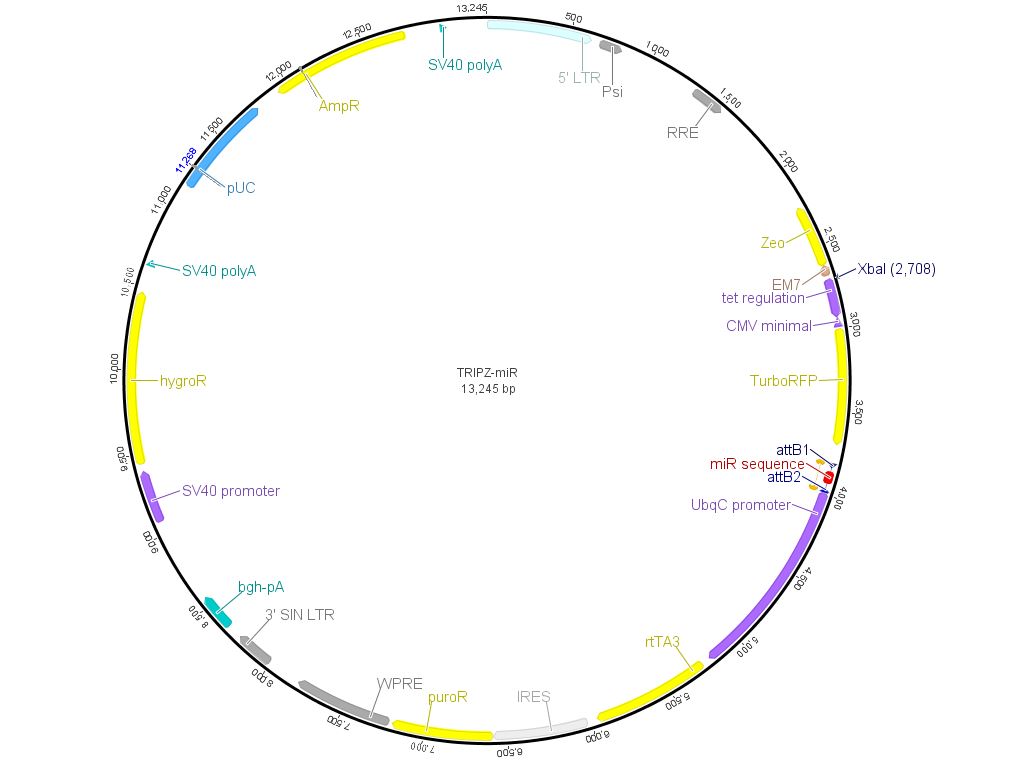


**Figure S8. Full length blots of whole-protein lysates probed with antibodies to WT1 and HSP90.**

Immunoblot of whole-protein lysates (40µg) probed with antibodies to WT1 and HSP90, as a loading control. The samples derive from the same experiment and were run on the same gel; the membrane was then cut in two and processed in parallel.

Lane 1=MDA-MB-231 GIPZ-lacZ

Lane 2=MDA-MB-231 GIPZ-WT1

Lane 3=MDA-MB-157 GIPZ-lacZ

Lane 4=MDA-MB-157 GIPZ-WT1

1 2 3 4 1 2 3 4


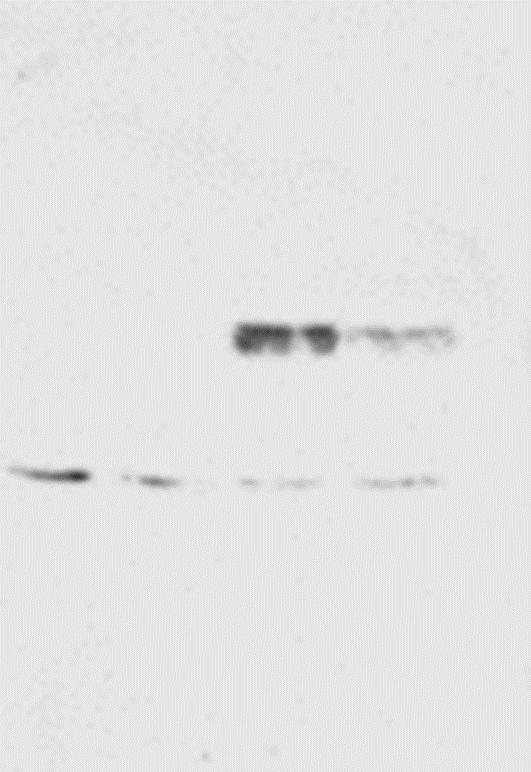

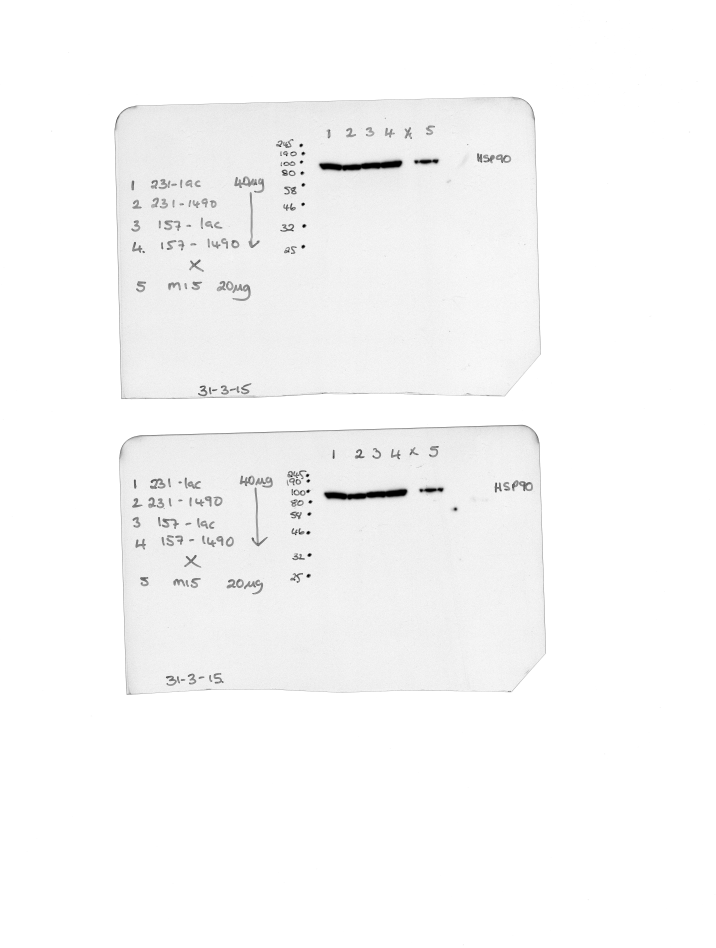


WT1

HSP90

**Additional file 2**

**Table S1. Significantly modulated genes identified through RNA-sequencing (fold change relative to WT1 kd vs lacZ).**

**Table S2. ToppFun functional enrichment analysis of the RNA-seq data**

**Table S3. List of the differentially expressed genes between *WT1*-positive and *WT1*-negative breast tumours (log2 Fold changes).**

**Table S4. Sequence of the shRNAs used in the knockdown study.**

**Table S5. Antibodies used for CSCs analysis.**

**Table S6. Primers and UPL probes used in the q-RT PCR experiments.**
